# Supplementary material for: Smooth muscle NF90 deficiency ameliorates diabetic atherosclerotic calcification in male mice via FBXW7-AGER1-AGEs axis
Source: Nat Commun. 2024 Jun 11;15:4985. doi: 10.1038/s41467-024-49315-9 (PMC11166998; doi:10.1038/s41467-024-49315-9)
Supplement: Supplementary file 1 — Supplementary Information [file 41467_2024_49315_MOESM1_ESM.pdf]

SUPPLEMENTARY INFORMATION

Smooth muscle NF90 deficiency ameliorates diabetic atherosclerotic calcification in male mice via FBXW7-AGER1-AGEs axis

Xie et al.

Figure.S1

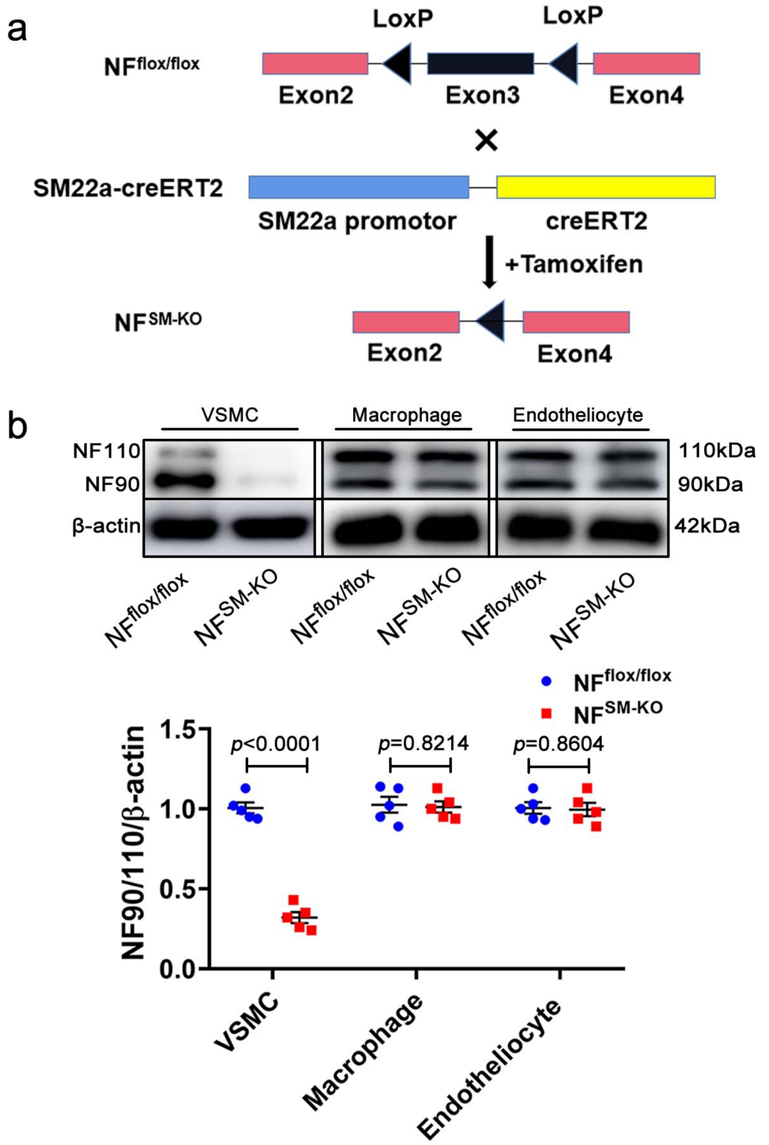

Supplementary Fig. 1 ***NF***$^{SM\text{-}KO}$ **mice were constructed. a** Schematic diagram of transgenic mice used to generate *NF*$^{SM\text{-}KO}$ mice. **b** Western blot analysis of NF90/110 protein levels in primary VSMCs, macrophages, and endotheliocytes (n=5 per group). Data were presented as mean ± SEM. Two-tailed Student unpaired *t* test for **b**. Source data are provided as a Source Data file.

Figure.S2

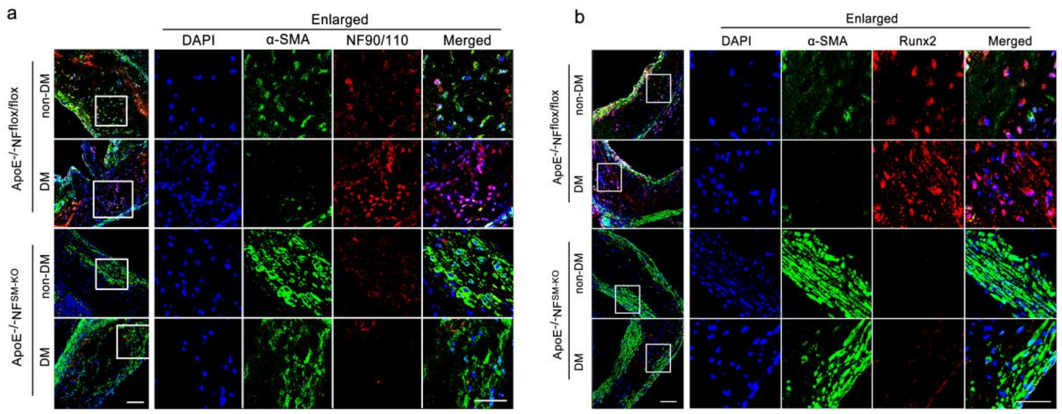

Supplementary Fig. 2 VSMC NF90/110 knockout inhibits diabetic atherosclerotic calcification. Immunofluorescence staining to analyze the expression of (**a**) α-SMA (green) and NF90/110 (red), (**b**) α-SMA (green) and Runx2 (red) in aortic roots of *ApoE$^{-/-}$NF$^{flox/flox}$* and *ApoE$^{-/-}$NF$^{SM\text{-}KO}$* mice with or without diabetes mellitus (DM; Scale bar: 50 μm, n=10 per group). Nuclei were stained with DAPI (blue).

Figure.S3

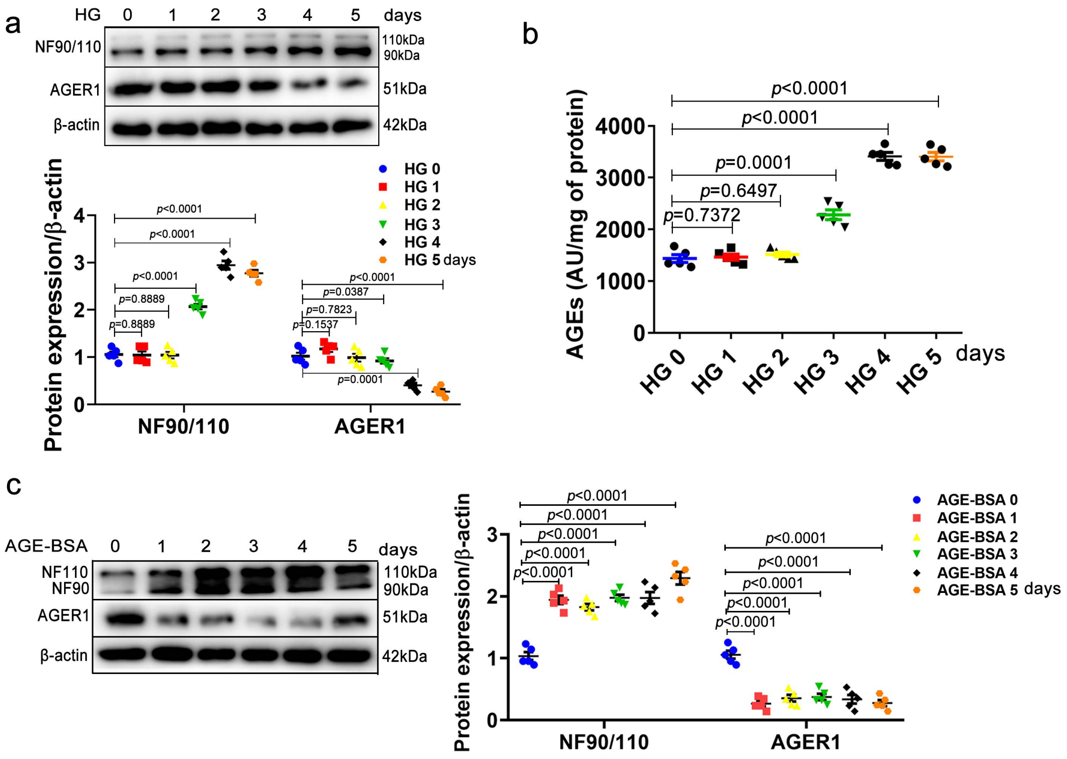

Supplementary Figure. 3 HG and AGEs increase NF90/110 and decrease AGER1 protein levels in HAVSMCs. a Western blot and quantification of NF90 / NF110 and AGER1 in HAVSMCs that were incubated in osteogenic medium with high glucose (HG; 27.5 mM glucose) for zero to five days (n=5 per group). **b** The AGEs levels in HG (27.5 mM glucose) osteogenic medium which cultured HAVSMCs for zero to five days were tested (n=5 per group). **c** Western blot and quantification of NF90/NF110 and AGER1 in HAVSMCs that were incubated in osteogenic medium with AGEs (200 μg/ml) for zero to five days (n=5 per group). BSA: bovine serum albumin. Data were presented as mean ± SEM. One-way ANOVA followed by Tukey's post-test analysis for **a-c**. *p*-values were adjusted for comparisons of multiple means. Source data are provided as a Source Data file.

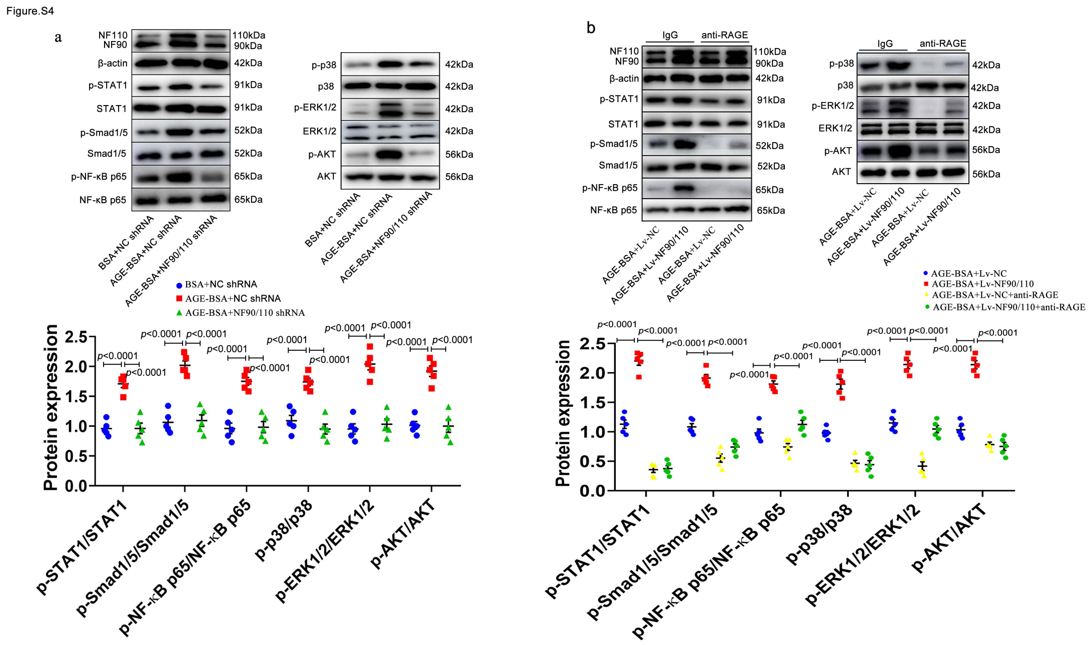

Supplementary Figure. 4 NF90/110 up-regulates AGE/RAGE-mediated signal pathways involved in diabetic atherosclerotic calcification. a Western blot and

quantification of phosphorylated protein levels of STAT1, Smad1/5, NF-κB p65, p38, ERK1/2, and AKT in HAVSMCs that were transfected with NF90/110 shRNA for NF90/110 silencing or NC shRNA, and treated with or without AGEs (200 µg/ml) for 24 hours (n=5 per group). BSA: bovine serum albumin. **b** HAVSMCs were transfected with lentivirus-NF90/110 (Lv-NF90/110) for NF90/110 overexpression or Lv-NC, and treated with or without AGEs (200 µg/ml), and then incubated with anti-RAGE antibody (20 µg/ml) or IgG for 24 hours. Western blot and quantification of phosphorylated protein levels of STAT1, Smad1/5, NF-κB p65, p38, ERK1/2, and AKT (n=5 per group). Data were presented as mean ± SEM. One-way ANOVA followed by Tukey's post-test analysis for **a-b**. *p*-values were adjusted for comparisons of multiple means. Source data are provided as a Source Data file.

Figure.S5

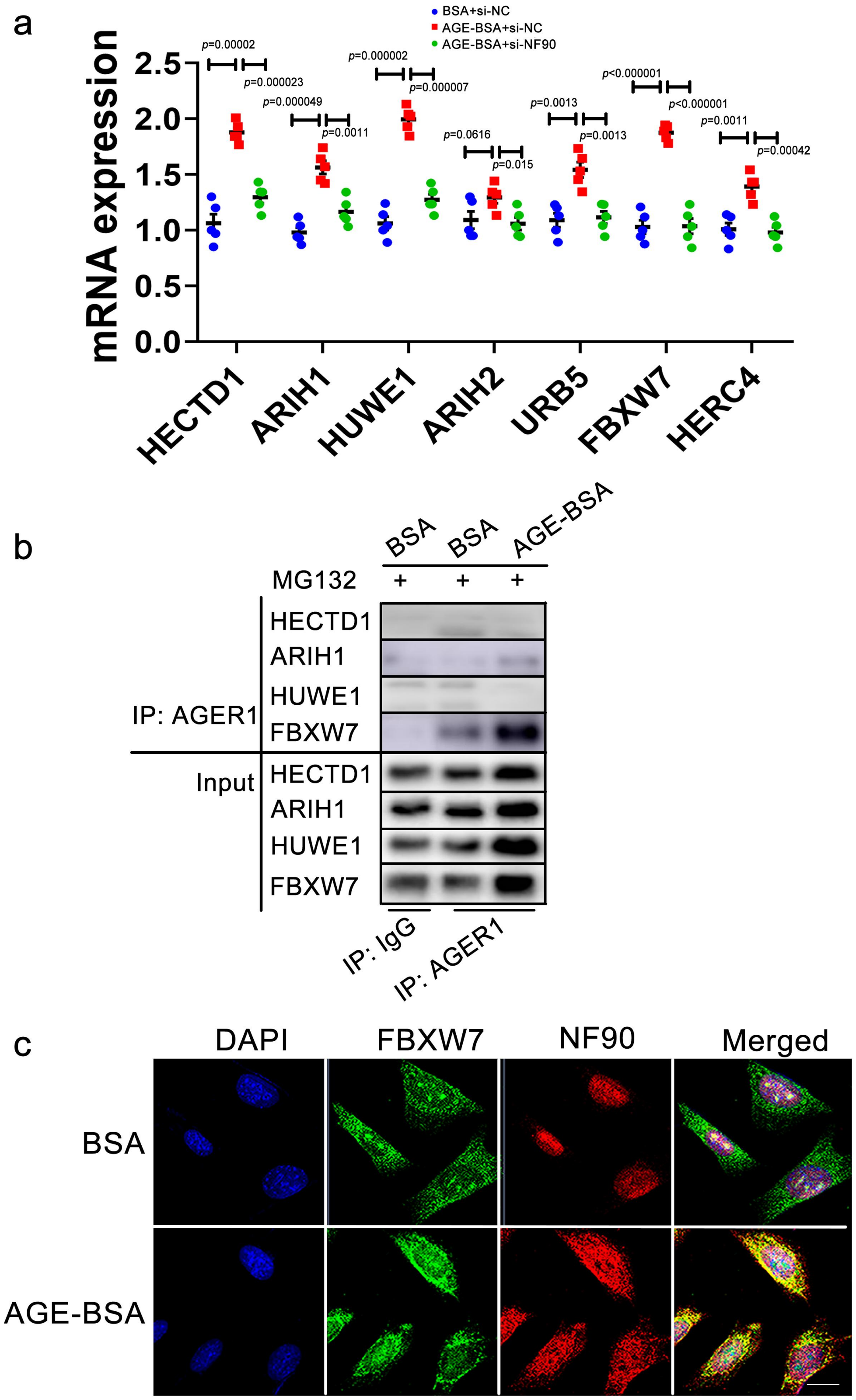

Supplementary Fig. 5 NF90 mediates AGE-induced up-regulation of FBXW7

expression. a HAVSMCs were transfected with NF90 siRNA (si-NF90) or NC siRNA and cultured in osteogenic medium with or without AGEs (200 μg/ml) for 24 hours. BSA: bovine serum albumin. The mRNA levels of HECTD1, ARIH1, HUWE1, ARIH2, URB5, FBXW7, and HERC4 are displayed (n=5 per group). **b** HAVSMCs were incubated in osteogenic medium with AGEs (200 μg/ml) and then treated with MG132 (10 μM) for 24 hours. Co-immunoprecipitation (Co-IP) and immunoblotting assays were performed to test the protein levels of HECTD1, ARIH1, HUWE1, and FBXW7 in interaction with AGER1 (n=5 per group). **c** Immunofluorescence double staining of FBXW7 (green) and NF90 (red) in HAVSMCs that were incubated in osteogenic medium with AGEs (200 μg/ml) for 24 hours. Nuclei were stained with DAPI (blue), and yellow indicates the co-localization of both proteins (Scale bar: 10 μm, n=5 per group). Data were presented as mean ± SEM. One-way ANOVA followed by Tukey's post-test analysis for **a**. *p*-values were adjusted for comparisons of multiple means. Source data are provided as a Source Data file.

Figure.S6

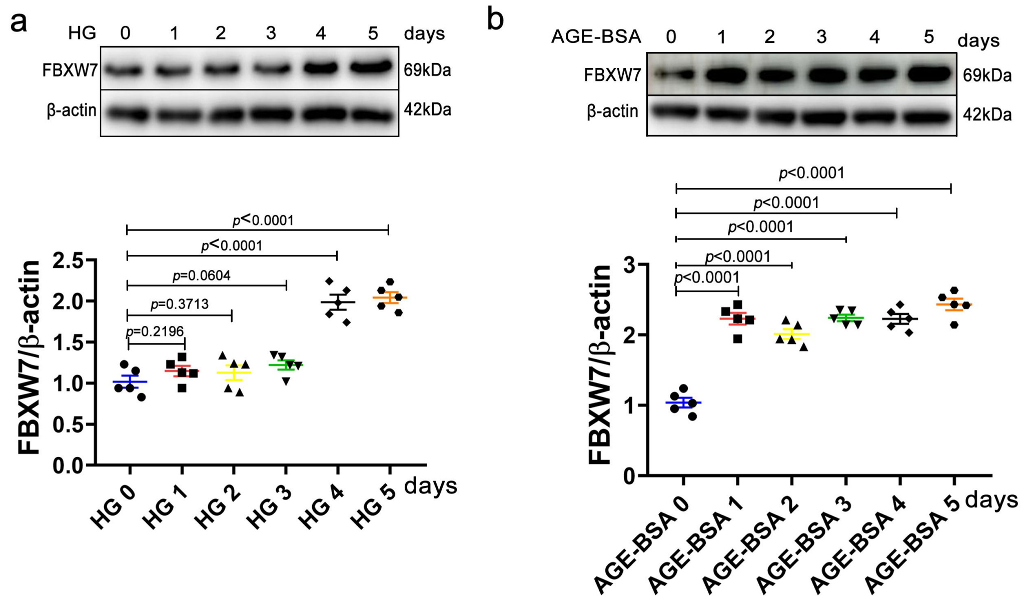

Supplementary Figure. 6 Both chronic HG and AGEs stimulation up-regulates FBXW7 expression in HAVSMCs. a Representative images of western blot and analysis of the level of FBXW7 in HAVSMCs that were incubated in osteogenic medium with high glucose (HG; 27.5 mM glucose) for zero to five days (n=5 per group). **b** Images of western blot and analysis of the level of FBXW7 in HAVSMCs that were incubated in osteogenic medium with AGEs (200 μg/ml) for zero to five days (n=5 per group). BSA: bovine serum albumin. Data were presented as mean ± SEM. One-way ANOVA followed by Tukey's post-test analysis for **a-b**. *p*-values were adjusted for comparisons of multiple means. Source data are provided as a Source Data file.

Figure.S7

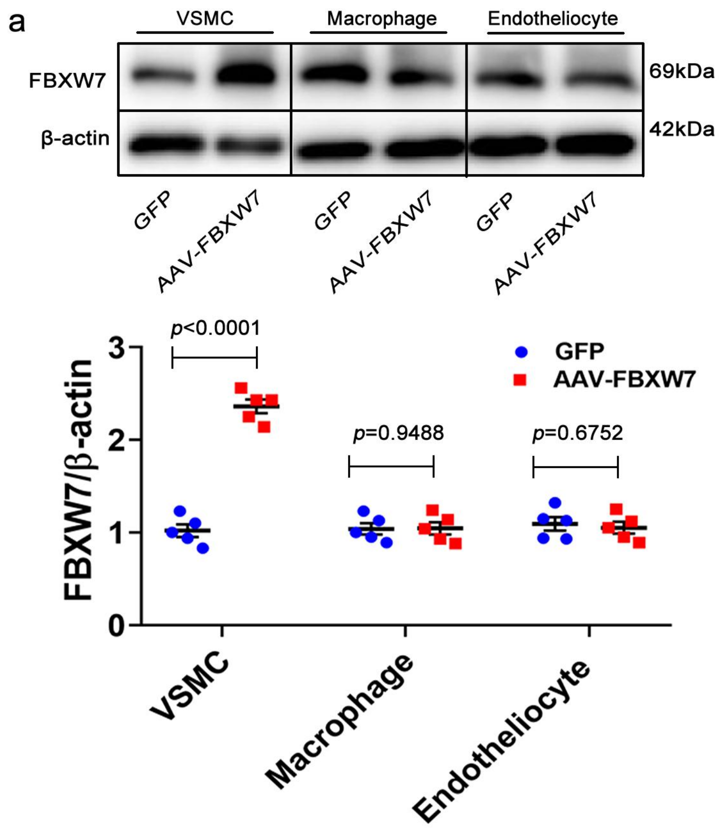

Supplementary Fig. 7 Transfection efficiency and specificity of adeno-associated viruses overexpressing FBXW7 in VSMCs. a Primary VSMCs, macrophages, and aortic endotheliocytes were isolated from mice after four weeks injected with AAV-GFP control or an AAV-FBXW7 vector. The protein levels of FBXW7 were tested by western blot in VSMCs, macrophages, and aortic endotheliocytes (n=10 per group). Data were presented as mean ± SEM. Two-tailed Student unpaired *t* test for **a**. Source data are provided as a Source Data file.

Supplementary table 1. Serum indices and body weight of mice in each group at the end of the experiment.

	$ApoE^{-/-}NF^{flox/flox}$	DM+$ApoE^{-/-}$ $NF^{flox/flox}$ ($^{*}p$ value)	$ApoE^{-/-}NF^{SM\text{-}KO}$ ($^{*}p$ value)	DM+$ApoE^{-/-}NF^{SM\text{-}KO}$ ($^{*}p$; $^{\#}p$ value)
BW (g)	28.58±0.72	24.16±0.7 (0.0006)	27.87±0.64 (0.8977)	24.15±0.75 (0.0006; >0.9999)
BG (mmol/l)	5.99±0.48	19.62±0.73 (<0.0001)	5.67±0.53 (0.9819)	19.93±0.67 (<0.0001; 0.9833)
TC (mmol/l)	22.52±0.89	23.63±0.90 (0.8161)	23.40±0.84 (0.8978)	22.81±0.92 (0.9958; 0.9142)
TG (mmol/l)	1.73±0.08	1.70±0.10 (0.9961)	1.72±0.09 (0.9996)	1.89±0.13 (0.6841; 0.5479)
HDL-C (mmol/L)	1.85±0.11	1.91±0.11 (09723)	1.95±0.07 (0.8773)	1.91±0.09 (0.9650; >0.9999)
LDL-C (mmol/L)	4.54±0.14	4.71±0.12 (0.7772)	4.64±0.12 (0.9391)	4.53±0.12 (>0.9999; 0.7457)
Calcium (mmol/l)	2.32±0.09	2.28±0.08 (0.9862)	2.20±0.07 (0.6785)	2.21±0.07 (0.7489; 0.9105)
Phosphorus (mmol/l)	3.43±0.08	3.34±0.11 (0.8718)	3.53±0.08 (0.8786)	3.4±0.09 (0.9936; 0.7383)
FINS (mmol/l)	13.41±0.79	5.68±0.61 (<0.0001)	11.97±0.7 (0.4373)	5.41±0.57 (<0.0001; 0.9914)

Data are presented as mean ± SEM, n=10 in each group. One-way ANOVA followed by Tukey's post-test analysis were used. *p*-values were adjusted for comparisons of multiple means. $p<0.05$ was statistically significant. $^{*}p<0.05$, vs. $ApoE^{-/-}NF^{flox/flox}$. $^{\#}p<0.05$, vs. DM+$ApoE^{-/-}NF^{flox/flox}$. Abbreviations: BW, body weight; BG, blood glucose; TC, total cholesterol; TG, triglycerides; HDL-C, high-density lipoprotein-C; LDL-C, low-density lipoprotein-C; FINS, fasting serum insulin.

Supplementary table 2. Serum indices and body weight of mice in each group at the end of the experiment.

	DM+$ApoE^{-/-}$ $NF^{flox/flox}$	DM+$ApoE^{-/-}$ $NF^{SM\text{-}KO}$ ($^{*}p$ value)	DM+$ApoE^{-/-}$ $NF^{flox/flox}$+Ad-FBXW7 ($^{*}p$ value)	DM+$ApoE^{-/-}$ $NF^{SM\text{-}KO}$+Ad-FBXW7 ($^{*}p$; $^{\#}p$ value)
BW (g)	24.73±0.96	24.01±0.82 (0.9418)	23.75±0.77 (0.8698)	24.09±1.03 (0.9579; >0.9999)
BG (mmol/l)	20.0±0.86	20.70±0.63 (0.9164)	20.35±0.83 (0.9882)	19.78±0.72 (0.9970; 0.8312)
TC (mmol/l)	22.11±1.06	21.69±1.00 (0.9895)	22.84±0.84 (0.9488)	21.79±0.93 (0.9953; 0.9999)

TG (mmol/l)	1.83±0.09	1.88±0.08 (0.9808)	1.92±0.09 (0.8871)	1.99±0.10 (0.6110; 0.8299)
HDL-C (mmol/L)	1.91±0.06	2.02±0.08 (0.8072)	1.90±0.08 (0.9995)	2.00±0.09 (0.8786; 0.9987)
LDL-C (mmol/L)	4.83±0.15	4.84±0.11 (0.9997)	4.72±0.10 (0.9098)	4.77±0.10 (0.9875; 0.9749)
Calcium (mmol/l)	2.31±0.09	2.26±0.11 (0.9833)	2.12±0.11 (0.5737)	2.31±0.10 (>0.9999; 0.9860)
Phosphorus (mmol/l)	3.31±0.10	3.35±0.12 (0.9935)	3.26±0.13 (0.9931)	3.19±0.13 (0.9151; 0.7984)
FINS (mmol/l)	6.71±0.68	6.31±0.56 (0.9714)	7.11±0.62 (0.9734)	6.83±0.74 (0.9994; 0.9433)

Data are presented as mean ± SEM, n=10 in each group. One-way ANOVA followed by Tukey's post-test analysis were used. *p*-values were adjusted for comparisons of multiple means. p<0.05 was statistically significant. $^{*}p$<0.05, vs. DM+$ApoE^{-/-}NF^{flox/flox}$. $^{\#}p$<0.05, vs. DM+$ApoE^{-/-}NF^{SM\text{-}KO}$. Abbreviations: BW, body weight; BG, blood glucose; TC, total cholesterol; TG, triglycerides; HDL-C, high-density lipoprotein-C; LDL-C, low-density lipoprotein-C; FINS, fasting serum insulin.

Supplementary table 3. Serum indices and body weights of patients with or without DM

	non-DM human specimens	DM human specimens	*p value*
Age (year)	38.50±1.13	39.60±1.23	0.5148
BW (kg)	76.30±1.94	69.60±1.25	0.0094
BG (mmol/l)	5.19±0.44	9.98±0.58	<0.0001
HbA1c	5.33±0.47	7.83±0.39	0.0006
TC (mmol/l)	3.55±0.21	3.82±0.26	0.4271
TG (mmol/l)	1.61±0.07	1.76±0.10	0.2139
HDL (mmol/L)	1.08±0.09	1.01±0.09	0.5814
LDL (mmol/L)	2.16±0.12	2.10±0.08	0.6868
Calcium (mmol/l)	2.44±0.04	2.42±0.06	0.7638
Phosphorus (mmol/l)	1.08±0.07	1.11±0.06	0.7958
FINS (uIU/ml)	19.35±1.38	10.82±1.14	0.0002

Data are presented as mean ± SEM, n=10 in each group. Two-tailed Student unpaired *t* test was used. p <0.05 was statistically significant. Abbreviations: BW, body weight;

BG, blood glucose; HbA1c: glycosylated hemoglobin; TC, total cholesterol; TG, triglycerides; HDL, high-density lipoprotein; LDL, low-density lipoprotein; FINS, fasting serum insulin.

Supplementary table 4. Primer sequences for RT-qPCR and RIP assay.

	Forward primer (5′-3′)	Reverse primer (5′-3′)
NF90/110 Homo	AACAAAAAGGTGGCGAAGGC	CCAGGGTTATGTGGCTTAGCA
AGER1 Homo	GTGGAGACCATCAGTGCCTT	ACTCAATCCCGCACTCACTGA
FBXW7 Homo	CCACTGGGCTTGTACCATGT	GGTCCACTCCAGCTCTGAAA
HECTD1 Homo	AATGGCTACAGATGGGACAGG	GCTGGTAAGAAAGTGCGAGG
ARIH1 Homo	AAAGTCTCGAACACGCCAGA	TGTCCACATTCAAGGCCAGT
HUWE1 Homo	GTGCCGAAAAGAATGTCCGT	ACACTGGACTCCATCCATATCA
ARIH2 Homo	GTGTGTATGCAGTTTGTGCGA	CATGCAAGAGACTCCCACGC
URB5 Homo	CAGCTCAATGACAGGTTACGA	AGAAAGGCAGCATGATTTGGT
HERC4 Homo	GCATCCTTTGGGCAGCTAGG	ACACTGTTCCATCATCCAGAACA
β-actin Homo	CATGTACGTTGCTATCCAGGC	CTCCTTAATGTCACGCACGAT
RIP: FBXW7 Homo	CCAAGAGCCACAACTCAAGC	TTCCTCCATCATGTCAGGTTGT
