# Supplementary material for: Smooth muscle NF90 deficiency ameliorates diabetic atherosclerotic calcification in male mice via FBXW7-AGER1-AGEs axis
Source: Nat Commun. 2024 Jun 11;15:4985. doi: 10.1038/s41467-024-49315-9 (PMC11166998; doi:10.1038/s41467-024-49315-9)
Supplement: Supplementary file 3 — Reporting Summary [file 41467_2024_49315_MOESM3_ESM.pdf]

nature portfolio

Corresponding author(s): Ming-xiang Zhang, Yu-guo Chen and Yun Zhang

Last updated by author(s): Apr 2, 2024

Reporting Summary

Nature Portfolio wishes to improve the reproducibility of the work that we publish. This form provides structure for consistency and transparency in reporting. For further information on Nature Portfolio policies, see our Editorial Policies and the Editorial Policy Checklist.

Statistics

For all statistical analyses, confirm that the following items are present in the figure legend, table legend, main text, or Methods section.

n/a	Confirmed	
☐	☒	The exact sample size (*n*) for each experimental group/condition, given as a discrete number and unit of measurement
☐	☒	A statement on whether measurements were taken from distinct samples or whether the same sample was measured repeatedly
☐	☒	The statistical test(s) used AND whether they are one- or two-sided<br>*Only common tests should be described solely by name; describe more complex techniques in the Methods section.*
☒	☐	A description of all covariates tested
☐	☒	A description of any assumptions or corrections, such as tests of normality and adjustment for multiple comparisons
☐	☒	A full description of the statistical parameters including central tendency (e.g. means) or other basic estimates (e.g. regression coefficient) AND variation (e.g. standard deviation) or associated estimates of uncertainty (e.g. confidence intervals)
☐	☒	For null hypothesis testing, the test statistic (e.g. *F*, *t*, *r*) with confidence intervals, effect sizes, degrees of freedom and *P* value noted<br>*Give P values as exact values whenever suitable.*
☒	☐	For Bayesian analysis, information on the choice of priors and Markov chain Monte Carlo settings
☒	☐	For hierarchical and complex designs, identification of the appropriate level for tests and full reporting of outcomes
☒	☐	Estimates of effect sizes (e.g. Cohen's *d*, Pearson's *r*), indicating how they were calculated

Our web collection on statistics for biologists contains articles on many of the points above.

Software and code

Policy information about availability of computer code

Data collection	ZEN 2009 Light Edition software; Amersham Image 680; LightCycler 480; Pannoramic Scanner; Leica H-700FA
Data analysis	Image J v18.0; SPSS 23.0; GraphPad Prism 8; FlowJo V10; Image Pro Plus 6.0

For manuscripts utilizing custom algorithms or software that are central to the research but not yet described in published literature, software must be made available to editors and reviewers. We strongly encourage code deposition in a community repository (e.g. GitHub). See the Nature Portfolio guidelines for submitting code & software for further information.

Data

Policy information about availability of data

All manuscripts must include a data availability statement. This statement should provide the following information, where applicable:

- Accession codes, unique identifiers, or web links for publicly available datasets
- A description of any restrictions on data availability
- For clinical datasets or third party data, please ensure that the statement adheres to our policy

RIP-seq data have been deposited to the NCBI Gene Expression Omnibus (GEO; GSE253682; https://www.ncbi.nlm.nih.gov/geo/guery/acc.cgi?acc=GSE253682).The proteome-seq data have been deposited to iProX (PXD048707; https://proteomecentral.proteomexchange.org/cgi/GetDataset?ID=PXD048707). Data supporting the findings of this study are available within the article and its Supplementary Information files. All the datasets presented in the paper are available from the corresponding author on request. Source data are provided with this paper.

Research involving human participants, their data, or biological material

Policy information about studies with human participants or human data. See also policy information about sex, gender (identity/presentation), and sexual orientation and race, ethnicity and racism.

Reporting on sex and gender	Participants were Chinese male. Based on the considerable evidence suggesting that estrogen modulates cardiovascular physiology and function in both health and disease, and that it could potentially serve as a cardioprotective agent. We considered the need to exclude the effect of gender at the beginning design of study and chose the specimens from male patients for this current study.
Reporting on race, ethnicity, or other socially relevant groupings	Race and ethnicity were not linked to the studies.
Population characteristics	Atherosclerotic epicardial coronary artery segments were collected from human specimens with or without DM (n=10 per group). Ten patients with DM with an average of 40 were included in this study. Ten patients without DM with average of 39 were included in this study. The specimens were donated by the Shandong Red Cross Society.
Recruitment	The specimens were donated by the Shandong Red Cross Society.
Ethics oversight	The experiment protocols were examined and approved by the review committee of Qilu Hospital of Shandong University, Jinan, China (ethics approval No. KYLL-2018(KS)-233).

Note that full information on the approval of the study protocol must also be provided in the manuscript.

Field-specific reporting

Please select the one below that is the best fit for your research. If you are not sure, read the appropriate sections before making your selection.

[x] Life sciences [ ] Behavioural & social sciences [ ] Ecological, evolutionary & environmental sciences

For a reference copy of the document with all sections, see nature.com/documents/nr-reporting-summary-flat.pdf

Life sciences study design

All studies must disclose on these points even when the disclosure is negative.

Sample size	Due to the novelty of the hypothesis, power analysis is based on speculation and on preliminary data, pertinent to specific endpoints. The N number for all experiments, including animal experiments and in vitro experiments were listed in the figure legends.
Data exclusions	No data were excluded from the analysis.
Replication	All in vivo and in vitro experiments were highly reproducible and were independently repeated at least 3 times. All experiments were performed independently multiple times using biologically independent replicated.
Randomization	Mice were randomly assigned to groups. Cells were grown under the same conditions and randomly allocated into different groups without any bias.
Blinding	The investigators were blinded to group allocation during data collection and analysis. We collected and analyzed the compared samples under the same conditions.

Behavioural & social sciences study design

All studies must disclose on these points even when the disclosure is negative.

Study description	*Briefly describe the study type including whether data are quantitative, qualitative, or mixed-methods (e.g. qualitative cross-sectional, quantitative experimental, mixed-methods case study).*
Research sample	*State the research sample (e.g. Harvard university undergraduates, villagers in rural India) and provide relevant demographic information (e.g. age, sex) and indicate whether the sample is representative. Provide a rationale for the study sample chosen. For studies involving existing datasets, please describe the dataset and source.*
Sampling strategy	*Describe the sampling procedure (e.g. random, snowball, stratified, convenience). Describe the statistical methods that were used to predetermine sample size OR if no sample-size calculation was performed, describe how sample sizes were chosen and provide a rationale for why these sample sizes are sufficient. For qualitative data, please indicate whether data saturation was considered, and what criteria were used to decide that no further sampling was needed.*

Data collection	*Provide details about the data collection procedure, including the instruments or devices used to record the data (e.g. pen and paper, computer, eye tracker, video or audio equipment) whether anyone was present besides the participant(s) and the researcher, and whether the researcher was blind to experimental condition and/or the study hypothesis during data collection.*
Timing	*Indicate the start and stop dates of data collection. If there is a gap between collection periods, state the dates for each sample cohort.*
Data exclusions	*If no data were excluded from the analyses, state so OR if data were excluded, provide the exact number of exclusions and the rationale behind them, indicating whether exclusion criteria were pre-established.*
Non-participation	*State how many participants dropped out/declined participation and the reason(s) given OR provide response rate OR state that no participants dropped out/declined participation.*
Randomization	*If participants were not allocated into experimental groups, state so OR describe how participants were allocated to groups, and if allocation was not random, describe how covariates were controlled.*

Ecological, evolutionary & environmental sciences study design

All studies must disclose on these points even when the disclosure is negative.

Study description	*Briefly describe the study. For quantitative data include treatment factors and interactions, design structure (e.g. factorial, nested, hierarchical), nature and number of experimental units and replicates.*
Research sample	*Describe the research sample (e.g. a group of tagged Passer domesticus, all Stenocereus thurberi within Organ Pipe Cactus National Monument), and provide a rationale for the sample choice. When relevant, describe the organism taxa, source, sex, age range and any manipulations. State what population the sample is meant to represent when applicable. For studies involving existing datasets, describe the data and its source.*
Sampling strategy	*Note the sampling procedure. Describe the statistical methods that were used to predetermine sample size OR if no sample-size calculation was performed, describe how sample sizes were chosen and provide a rationale for why these sample sizes are sufficient.*
Data collection	*Describe the data collection procedure, including who recorded the data and how.*
Timing and spatial scale	*Indicate the start and stop dates of data collection, noting the frequency and periodicity of sampling and providing a rationale for these choices. If there is a gap between collection periods, state the dates for each sample cohort. Specify the spatial scale from which the data are taken*
Data exclusions	*If no data were excluded from the analyses, state so OR if data were excluded, describe the exclusions and the rationale behind them, indicating whether exclusion criteria were pre-established.*
Reproducibility	*Describe the measures taken to verify the reproducibility of experimental findings. For each experiment, note whether any attempts to repeat the experiment failed OR state that all attempts to repeat the experiment were successful.*
Randomization	*Describe how samples/organisms/participants were allocated into groups. If allocation was not random, describe how covariates were controlled. If this is not relevant to your study, explain why.*
Blinding	*Describe the extent of blinding used during data acquisition and analysis. If blinding was not possible, describe why OR explain why blinding was not relevant to your study.*

Did the study involve field work? ☐ Yes ☐ No

Field work, collection and transport

Field conditions	*Describe the study conditions for field work, providing relevant parameters (e.g. temperature, rainfall).*
Location	*State the location of the sampling or experiment, providing relevant parameters (e.g. latitude and longitude, elevation, water depth).*
Access & import/export	*Describe the efforts you have made to access habitats and to collect and import/export your samples in a responsible manner and in compliance with local, national and international laws, noting any permits that were obtained (give the name of the issuing authority, the date of issue, and any identifying information).*
Disturbance	*Describe any disturbance caused by the study and how it was minimized.*

Reporting for specific materials, systems and methods

We require information from authors about some types of materials, experimental systems and methods used in many studies. Here, indicate whether each material, system or method listed is relevant to your study. If you are not sure if a list item applies to your research, read the appropriate section before selecting a response.

Materials & experimental systems

n/a	Involved in the study	
☐	☒	Antibodies
☐	☒	Eukaryotic cell lines
☒	☐	Palaeontology and archaeology
☐	☒	Animals and other organisms
☒	☐	Clinical data
☒	☐	Dual use research of concern
☒	☐	Plants

Methods

n/a	Involved in the study	
☒	☐	ChIP-seq
☐	☒	Flow cytometry
☒	☐	MRI-based neuroimaging

Antibodies

Antibodies used

1. β-actin, RRID: AB_3083534, Sigma, Catalog number: ZRB1312. Host organism: rabbit. Target antigen: Mouse, Monkey, Rat, Human. Applications: ICC, IHC (p), WB.
2. HUWE1, RRID: AB_3083532, Abcam, Catalog number: ab271032. Host organism: rabbit. Target antigen: Mouse, Rat, Human. Applications: Flow Cyt (Intra), ICC/IF, IHC-P, WB.
3. ILF3, RRID:AB_2049804, Abcam, Catalog number: ab92355. Host organism: rabbit. Target antigen: Mouse, Rat, Human. Applications: WB, IHC-P, Flow Cyt (Intra), ICC/IF.
4. Msx2, RRID: AB_3083530, Abcam, Catalog number: ab223692. Host organism: rabbit. Target antigen: Human. Applications: IHC-P.
5. Runx2: RRID: AB_2713945, Abcam, Catalog number: ab192256. Host organism: rabbit. Target antigen: Mouse, Human. Applications: IHC-P, ICC/IF, Flow Cyt (Intra), ChIC/CUT&RUN-seq.
6. FBXW7: RRID: AB_2687519, Abcam, Catalog number: ab109617. Host organism: rabbit. Target antigen: Human. Applications: IHC-P, WB, ICC/IF, IP.
7. OPN: RRID: AB_306566, Abcam, Catalog number: ab8448. Host organism: rabbit. Target antigen: Mouse, Rat, Human. Applications: Flow Cyt (Intra), ICC/IF, IHC-P, WB.BAx
8. Bax: RRID: AB_725631, Abcam, Catalog number: ab32503. Host organism: rabbit. Target antigen: Mouse, Rat, Human. Applications: IHC-P, IP, sELISA, WB.
9. NF90, RRID: AB_3083533, Santa cruz, Catalog number: sc-377406. Host organism: mouse. Target antigen: Human, Mouse, Rat. Applications: WB, IF, ELISA, IHC, IP.
10. DDOST (AGER1), RRID: AB_2230534, Proteintech, Catalog number: 14916-1-AP. Host organism: rabbit. Target antigen: Human, Mouse, Rat. Applications: WB, ELISA, IHC.
11. AGEs, RRID: AB_447638, Abcam, Catalog number: ab23722. Host organism: rabbit. Target antigen: Species independent. Applications: ELISA, ICC/IF, IHC-Fr, IHC-P, WB.
12. OST48 (AGER1), RRID: AB_1125745, Santa cruz, Catalog number: sc-74408. Host organism: mouse. Target antigen: Human, Mouse, Rat. Applications: WB, IF, ELISA, IHC, IP.
13. OPN, RRID: AB_2194997, Santa cruz, Catalog number: sc-21742. Host organism: mouse. Target antigen: Human, Mouse, Rat. Applications: WB, IF, IHC, IP.
14. α-SMA, RRID: AB_262054, Sigma, Catalog number: A5228. Host organism: mouse. Target antigen: human, mouse, rat, chicken, frog, canine, rabbit, guinea pig, goat, bovine, sheep, snake. Applications: ARR, ELISA (i), ICC, IF, IHC (p), WB.
15. Msx2, RRID: AB_3083529, Abcam, Catalog number: ab227720. Host organism: rabbit. Target antigen: Mouse, Human. Applications: WB.
16. Runx2, RRID: AB_2732805, Cell Signalling Technology, Catalog number: 12556. Host organism: rabbit. Target antigen: human, mouse, rat. Applications: IP, F, CHIP, IF, WB.
17. BMP2, RRID: AB_2227877, Novus, Catalog number: NBP1-19751. Host organism: mouse. Target antigen: Human, Mouse, Rat, Canine. Applications: WB, ICC/IF, IHC.
18. calponin, RRID: AB_2291941, Abcam, Catalog number: ab46794. Host organism: rabbit. Target antigen: Mouse, Rat, Human, Pig. Applications: ICC/IF, IHC-P, WB.
19. Bax, RRID: AB_10695870, Cell Signalling Technology, Catalog number: 2772S. Host organism: rabbit. Target antigen: human, mouse, rat, monkey. Applications: IP, WB.
20. Bcl-2, RRID: AB_2835021, Affinity biosciences, Catalog number: AF6139. Host organism: rabbit. Target antigen: Human, Mouse, Rat, Chinese Mitten Crab. Applications: WB, IHC, ICC/IF.
21. Cleaved-Caspase-3, RRID: AB_302962, Abcam, Catalog number: ab2302. Host organism: rabbit. Target antigen: Human, Recombinant fragment. Applications: WB.
22. ubiquitin, RRID: AB_671515, Proteintech, Catalog number: 10201-2-AP),. Host organism: rabbit. Target antigen: Human, Mouse, Rat. Applications: ChIP, CoIP, IF, IHC, WB.
23. HA-tag, RRID: AB_1549585, Cell Signalling Technology, Catalog number: 3724. Host organism: rabbit. Target antigen: Species independent. Applications: IP, WB, IHC, IF, F, CHIP.
24. Myc-Tag, RRID: AB_490778, Cell Signalling Technology, Catalog number: 2278. Host organism: rabbit. Target antigen: Species independent. Applications: IP, WB, IF, F.
25. Flag-Tag, RRID: AB_2916341, Abcam, Catalog number: ab205606. Host organism: rabbit. Target antigen: Species independent. Applications: Flow Cyt, ICC/IF, IHC-P, IP, WB.
26. p-STAT1, RRID: AB_561284, Cell Signalling Technology, Catalog number: 9167. Host organism: rabbit. Target antigen: Human, Mouse. Applications: IP, WB, IHC, IF, F, CHIP.
27. STAT1, RRID: AB_2737027, Cell Signalling Technology, Catalog number: 14994. Host organism: rabbit. Target antigen: Human,

Mouse, Rat, Monkey. Applications: IP, WB, IHC, IF, F, CHIP, C&R.
28. p-smad1/5, RRID: AB_491015, Cell Signalling Technology, Catalog number: 9516. Host organism: rabbit. Target antigen: Human, Mouse, Rat. Applications: WB, IF, F.
29. Smad1/5, RRID: AB_3083531, Abcam, Catalog number: ab300164. Host organism: rabbit. Target antigen: Mouse, Rat, Human. Applications: WB.
30. p-NF-κB p65, RRID: AB_331284, Cell Signalling Technology, Catalog number: 3033S. Host organism: rabbit. Target antigen: Human, Mouse, Rat, Monkey, Bovine, Dog, Mink, Pig, Hamster. Applications: IP, WB, IF, F.
31. NF-κB p65, RRID: AB_10828935, Cell Signalling Technology, Catalog number: 6956S. Host organism: mouse. Target antigen: Human, Mouse, Rat, Monkey, Pig, Hamster. Applications: IP, WB, IF, F, IHC, CHIP.
32. p-p38, RRID: AB_2139682, Cell Signalling Technology, Catalog number: 4511S. Host organism: rabbit. Target antigen: Human, Mouse, Rat, Monkey, Mink, Pig, S. cerevisiae. Applications: IP, WB, IF, F, IHC.
33. p38, RRID: AB_10999090, Cell Signalling Technology, Catalog number: 8690S. Host organism: rabbit. Target antigen: Human, Mouse, Rat, Hamster, Monkey, Bovine, Pig. Applications: WB, IF, F, IHC.
34. p-ERK1/2, RRID: AB_2315112, Cell Signalling Technology, Catalog number: 4370S. Host organism: rabbit. Target antigen: Human, Mouse, Rat, Hamster, Monkey, Mink, D. melanogaster, Zebrafish, Bovine, Dog, Pig, S. cerevisiae. Applications: WB, IF, F, IHC, IP.
35. ERK1/2, RRID: AB_390779, Cell Signalling Technology, Catalog number: 4695S. Host organism: rabbit. Target antigen: Human, Mouse, Rat, Hamster, Monkey, Mink, D. melanogaster, Zebrafish, Bovine, Dog, Pig, C. elegans. Applications: WB, IF, F, IHC, IP.
36. p-AKT, RRID: AB_2315049, Cell Signalling Technology, Catalog number: 4060S. Host organism: rabbit. Target antigen: Human, Mouse, Rat, Hamster, Monkey, D. melanogaster, Zebrafish, Bovine. Applications: WB, IF, F, IHC, IP.
37. AKT, RRID: AB_915783, Cell Signalling Technology, Catalog number: 4691S. Host organism: rabbit. Target antigen: Human, Mouse, Rat, Monkey, D. melanogaster. Applications: WB, IF, F, IHC, IP.
38. HECTD1, RRID: AB_10711075, Abcam, Catalog number: ab101992. Host organism: rabbit. Target antigen: Human, Pig. Applications: IP, WB.
39. ARIH1, RRID: AB_2768438, ABclonal, Catalog number: A17123. Host organism: rabbit. Target antigen: Human. Applications: WB.

Validation

1. β-actin, RRID: AB_3083534, Sigma, Catalog number: ZRB1312. Host organism: rabbit. Target antigen: Mouse, Monkey, Rat, Human. Applications: ICC, IHC (p), WB.
2. HUWE1, RRID: AB_3083532, Abcam, Catalog number: ab271032. Host organism: rabbit. Target antigen: Mouse, Rat, Human. Applications: Flow Cyt (Intra), ICC/IF, IHC-P, WB.
3. ILF3, RRID:AB_2049804, Abcam, Catalog number: ab92355. Host organism: rabbit. Target antigen: Mouse, Rat, Human. Applications: WB, IHC-P, Flow Cyt (Intra), ICC/IF.
4. Msx2, RRID: AB_3083530, Abcam, Catalog number: ab223692. Host organism: rabbit. Target antigen: Human. Applications: IHC-P.
5. Runx2: RRID: AB_2713945, Abcam, Catalog number: ab192256. Host organism: rabbit. Target antigen: Mouse, Human. Applications: IHC-P, ICC/IF, Flow Cyt (Intra), ChIC/CUT&RUN-seq.
6. FBXW7: RRID: AB_2687519, Abcam, Catalog number: ab109617. Host organism: rabbit. Target antigen: Human. Applications: IHC-P, WB, ICC/IF, IP.
7. OPN: RRID: AB_306566, Abcam, Catalog number: ab8448. Host organism: rabbit. Target antigen: Mouse, Rat, Human. Applications: Flow Cyt (Intra), ICC/IF, IHC-P, WB.BAx
8. Bax: RRID: AB_725631, Abcam, Catalog number: ab32503. Host organism: rabbit. Target antigen: Mouse, Rat, Human. Applications: IHC-P, IP, sELISA, WB.
9. NF90, RRID: AB_3083533, Santa cruz, Catalog number: sc-377406. Host organism: mouse. Target antigen: Human, Mouse, Rat. Applications: WB, IF, ELISA, IHC, IP.
10. DDOST (AGER1), RRID: AB_2230534, Proteintech, Catalog number: 14916-1-AP. Host organism: rabbit. Target antigen: Human, Mouse, Rat. Applications: WB, ELISA, IHC.
11. AGEs, RRID: AB_447638, Abcam, Catalog number: ab23722. Host organism: rabbit. Target antigen: Species independent. Applications: ELISA, ICC/IF, IHC-Fr, IHC-P, WB.
12. OST48 (AGER1), RRID: AB_1125745, Santa cruz, Catalog number: sc-74408. Host organism: mouse. Target antigen: Human, Mouse, Rat. Applications: WB, IF, ELISA, IHC, IP.
13. OPN, RRID: AB_2194997, Santa cruz, Catalog number: sc-21742. Host organism: mouse. Target antigen: Human, Mouse, Rat. Applications: WB, IF, IHC, IP.
14. α-SMA, RRID: AB_262054, Sigma, Catalog number: A5228. Host organism: mouse. Target antigen: human, mouse, rat, chicken, frog, canine, rabbit, guinea pig, goat, bovine, sheep, snake. Applications: ARR, ELISA (i), ICC, IF, IHC (p), WB.
15. Msx2, RRID: AB_3083529, Abcam, Catalog number: ab227720. Host organism: rabbit. Target antigen: Mouse, Human. Applications: WB.
16. Runx2, RRID: AB_2732805, Cell Signalling Technology, Catalog number: 12556. Host organism: rabbit. Target antigen: human, mouse, rat. Applications: IP, F, CHIP, IF, WB.
17. BMP2, RRID: AB_2227877, Novus, Catalog number: NBP1-19751. Host organism: mouse. Target antigen: Human, Mouse, Rat, Canine. Applications: WB, ICC/IF, IHC.
18. calponin, RRID: AB_2291941, Abcam, Catalog number: ab46794. Host organism: rabbit. Target antigen: Mouse, Rat, Human, Pig. Applications: ICC/IF, IHC-P, WB.
19. Bax, RRID: AB_10695870, Cell Signalling Technology, Catalog number: 2772S. Host organism: rabbit. Target antigen: human, mouse, rat, monkey. Applications: IP, WB.
20. Bcl-2, RRID: AB_2835021, Affinity biosciences, Catalog number: AF6139. Host organism: rabbit. Target antigen: Human, Mouse, Rat, Chinese Mitten Crab. Applications: WB, IHC, ICC/IF.
21. Cleaved-Caspase-3, RRID: AB_302962, Abcam, Catalog number: ab2302. Host organism: rabbit. Target antigen: Human, Recombinant fragment. Applications: WB.
22. ubiquitin, RRID: AB_671515, Proteintech, Catalog number: 10201-2-AP),. Host organism: rabbit. Target antigen: Human, Mouse, Rat. Applications: ChIP, CoIP, IF, IHC, WB.
23. HA-tag, RRID: AB_1549585, Cell Signalling Technology, Catalog number: 3724. Host organism: rabbit. Target antigen: Species independent. Applications: IP, WB, IHC, IF, F, CHIP.
24. Myc-Tag, RRID: AB_490778, Cell Signalling Technology, Catalog number: 2278. Host organism: rabbit. Target antigen: Species independent. Applications: IP, WB, IF, F.

25. Flag-Tag, RRID: AB_2916341, Abcam, Catalog number: ab205606. Host organism: rabbit. Target antigen: Species independent. Applications: Flow Cyt, ICC/IF, IHC-P, IP, WB.
26. p-STAT1, RRID: AB_561284, Cell Signalling Technology, Catalog number: 9167. Host organism: rabbit. Target antigen: Human, Mouse. Applications: IP, WB, IHC, IF, F, CHIP.
27. STAT1, RRID: AB_2737027, Cell Signalling Technology, Catalog number: 14994. Host organism: rabbit. Target antigen: Human, Mouse, Rat, Monkey. Applications: IP, WB, IHC, IF, F, CHIP, C&R.
28. p-smad1/5, RRID: AB_491015, Cell Signalling Technology, Catalog number: 9516. Host organism: rabbit. Target antigen: Human, Mouse, Rat. Applications: WB, IF, F.
29. Smad1/5, RRID: AB_3083531, Abcam, Catalog number: ab300164. Host organism: rabbit. Target antigen: Mouse, Rat, Human. Applications: WB.
30. p-NF-κB p65, RRID: AB_331284, Cell Signalling Technology, Catalog number: 3033S. Host organism: rabbit. Target antigen: Human, Mouse, Rat, Monkey, Bovine, Dog, Mink, Pig, Hamster. Applications: IP, WB, IF, F.
31. NF-κB p65, RRID: AB_10828935, Cell Signalling Technology, Catalog number: 6956S. Host organism: mouse. Target antigen: Human, Mouse, Rat, Monkey, Pig, Hamster. Applications: IP, WB, IF, F, IHC, CHIP.
32. p-p38, RRID: AB_2139682, Cell Signalling Technology, Catalog number: 4511S. Host organism: rabbit. Target antigen: Human, Mouse, Rat, Monkey, Mink, Pig, S. cerevisiae. Applications: IP, WB, IF, F, IHC.
33. p38, RRID: AB_10999090, Cell Signalling Technology, Catalog number: 8690S. Host organism: rabbit. Target antigen: Human, Mouse, Rat, Hamster, Monkey, Bovine, Pig. Applications: WB, IF, F, IHC.
34. p-ERK1/2, RRID: AB_2315112, Cell Signalling Technology, Catalog number: 4370S. Host organism: rabbit. Target antigen: Human, Mouse, Rat, Hamster, Monkey, Mink, D. melanogaster, Zebrafish, Bovine, Dog, Pig, S. cerevisiae. Applications: WB, IF, F, IHC, IP.
35. ERK1/2, RRID: AB_390779, Cell Signalling Technology, Catalog number: 4695S. Host organism: rabbit. Target antigen: Human, Mouse, Rat, Hamster, Monkey, Mink, D. melanogaster, Zebrafish, Bovine, Dog, Pig, C. elegans. Applications: WB, IF, F, IHC, IP.
36. p-AKT, RRID: AB_2315049, Cell Signalling Technology, Catalog number: 4060S. Host organism: rabbit. Target antigen: Human, Mouse, Rat, Hamster, Monkey, D. melanogaster, Zebrafish, Bovine. Applications: WB, IF, F, IHC, IP.
37. AKT, RRID: AB_915783, Cell Signalling Technology, Catalog number: 4691S. Host organism: rabbit. Target antigen: Human, Mouse, Rat, Monkey, D. melanogaster. Applications: WB, IF, F, IHC, IP.
38. HECTD1, RRID: AB_10711075, Abcam, Catalog number: ab101992. Host organism: rabbit. Target antigen: Human, Pig. Applications: IP, WB.
39. ARIH1, RRID: AB_2768438, ABclonal, Catalog number: A17123. Host organism: rabbit. Target antigen: Human. Applications: WB.

Eukaryotic cell lines

Policy information about cell lines and Sex and Gender in Research

Cell line source(s)	HAVSMCs and HEK293T cells were obtained from American Type Culture Collection (ATCC, Rockefeller, MD, USA).
Authentication	The cell lines were not authenticated.
Mycoplasma contamination	All cell lines tested negative for mycoplasma contamination.
Commonly misidentified lines (See ICLAC register)	No commonly misidentified cell lines are used in the study.

Palaeontology and Archaeology

Specimen provenance	*Provide provenance information for specimens and describe permits that were obtained for the work (including the name of the issuing authority, the date of issue, and any identifying information). Permits should encompass collection and, where applicable, export.*
Specimen deposition	*Indicate where the specimens have been deposited to permit free access by other researchers.*
Dating methods	*If new dates are provided, describe how they were obtained (e.g. collection, storage, sample pretreatment and measurement), where they were obtained (i.e. lab name), the calibration program and the protocol for quality assurance OR state that no new dates are provided.*

☐ Tick this box to confirm that the raw and calibrated dates are available in the paper or in Supplementary Information.

Ethics oversight	*Identify the organization(s) that approved or provided guidance on the study protocol, OR state that no ethical approval or guidance was required and explain why not.*

Note that full information on the approval of the study protocol must also be provided in the manuscript.

Animals and other research organisms

Policy information about studies involving animals; ARRIVE guidelines recommended for reporting animal research, and Sex and Gender in Research

Laboratory animals	All mice we used were all male mice of 6-8 weeks. The strains included NF90/NF110 knockout floxed (NFflox/flox) mice, SM22a-creERT2 mice, and ApoE-/- mice in a C57BL/6J background. The VSMC conditional NF90/NF110 knockout (NFSM-KO) mice were bred from NFflox/flox mice crossed with SM22a-creERT2 mice (NFflox/flox/Cre+). The ApoE-/-NFSM-KO double knockout mice were

	generated by crossing ApoE-/- mice and NFflox/flox/Cre+ mice. Mice were housed in a pathogen-free animals care facility with humidity of 40-60% at a constant temperature (24℃℃) and a conventional light/dark cycle (12/12 h) under free conditions.
Wild animals	The study did not involve wild animals.
Reporting on sex	Based on the considerable evidence suggesting that estrogen modulates cardiovascular physiology and function in both health and disease, and that it could potentially serve as a cardioprotective agent. We considered the need to exclude the effect of gender at the beginning design of study and chose the specimens from male mice for this current study. The animals we used were all male mice according to similar studies in the field, so the findings in vivo applied to only male mice.
Field-collected samples	The study did not involve samples collected from the field.
Ethics oversight	Institutional Animal Care and Use Committee of Shandong University

Note that full information on the approval of the study protocol must also be provided in the manuscript.

Clinical data

Policy information about clinical studies

All manuscripts should comply with the ICMJEguidelines for publication of clinical research and a completedCONSORT checklist must be included with all submissions.

Clinical trial registration	*Provide the trial registration number from ClinicalTrials.gov or an equivalent agency.*
Study protocol	*Note where the full trial protocol can be accessed OR if not available, explain why.*
Data collection	*Describe the settings and locales of data collection, noting the time periods of recruitment and data collection.*
Outcomes	*Describe how you pre-defined primary and secondary outcome measures and how you assessed these measures.*

Dual use research of concern

Policy information about dual use research of concern

Hazards

Could the accidental, deliberate or reckless misuse of agents or technologies generated in the work, or the application of information presented in the manuscript, pose a threat to:

No	Yes	
☐	☐	Public health
☐	☐	National security
☐	☐	Crops and/or livestock
☐	☐	Ecosystems
☐	☐	Any other significant area

Experiments of concern

Does the work involve any of these experiments of concern:

No	Yes	
☐	☐	Demonstrate how to render a vaccine ineffective
☐	☐	Confer resistance to therapeutically useful antibiotics or antiviral agents
☐	☐	Enhance the virulence of a pathogen or render a nonpathogen virulent
☐	☐	Increase transmissibility of a pathogen
☐	☐	Alter the host range of a pathogen
☐	☐	Enable evasion of diagnostic/detection modalities
☐	☐	Enable the weaponization of a biological agent or toxin
☐	☐	Any other potentially harmful combination of experiments and agents

Plants

Seed stocks | NA

Novel plant genotypes | NA

Authentication | NA

ChIP-seq

Data deposition

☐ Confirm that both raw and final processed data have been deposited in a public database such as GEO.

☐ Confirm that you have deposited or provided access to graph files (e.g. BED files) for the called peaks.

Data access links
May remain private before publication.
For "Initial submission" or "Revised version" documents, provide reviewer access links. For your "Final submission" document, provide a link to the deposited data.

Files in database submission
Provide a list of all files available in the database submission.

Genome browser session
(e.g. UCSC)
Provide a link to an anonymized genome browser session for "Initial submission" and "Revised version" documents only, to enable peer review. Write "no longer applicable" for "Final submission" documents.

Methodology

Replicates
Describe the experimental replicates, specifying number, type and replicate agreement.

Sequencing depth
Describe the sequencing depth for each experiment, providing the total number of reads, uniquely mapped reads, length of reads and whether they were paired- or single-end.

Antibodies
Describe the antibodies used for the ChIP-seq experiments; as applicable, provide supplier name, catalog number, clone name, and lot number.

Peak calling parameters
Specify the command line program and parameters used for read mapping and peak calling, including the ChIP, control and index files used.

Data quality
Describe the methods used to ensure data quality in full detail, including how many peaks are at FDR 5% and above 5-fold enrichment.

Software
Describe the software used to collect and analyze the ChIP-seq data. For custom code that has been deposited into a community repository, provide accession details.

Flow Cytometry

Plots

Confirm that:

☒ The axis labels state the marker and fluorochrome used (e.g. CD4-FITC).

☒ The axis scales are clearly visible. Include numbers along axes only for bottom left plot of group (a 'group' is an analysis of identical markers).

☒ All plots are contour plots with outliers or pseudocolor plots.

☒ A numerical value for number of cells or percentage (with statistics) is provided.

Methodology

Sample preparation
After exposure to different intervenes, HAVSMCs were double stained with PE Annexin V Apoptosis Detection Kits I (BD Biosciences, San Jose, CA, USA). Cells were harvested and resuspended in 100 uL of Annexin V binding buffer containing 5 µl PE Annexin V and 5 µl 7-Amino-Actinomycin for 15 min at room temperature in the dark, An amount of 400 ul Annexin v binding buffer was added to each tube.

Instrument
BD FACS Calibur (BD Biosciences, San Jose, CA, USA) was used to examine the fluorescence intensity

Software	All data were analyzed by FlowJo (version V10).
Cell population abundance	At least 20,000 events were acquired for cells in the defined gate.
Gating strategy	Cellular debris were excluded by setting exclusion gates based on FSC and SSC. An untreated sample was run to adjust the voltage and gain for the 7AAD and PE detectors so that all cells can be detected in the bottom left quadrant. A treated sample stained with PE alone was run to adjust the voltage and gain for the PE detector so that the dead cells appear in the bottom right quadrant. A treated sample stained with 7AAD alone was run to adjust the voltage and gain for the 7AAD detector so that the dead cells appear in the top left quadrant. A treated sample stained with PE and 7AAD was run to adjust the compensation so that the live cells appear in the bottom left, the apoptotic cells appear in the bottom right, and the necrotic cells appear in the top right quadrants.

☒ Tick this box to confirm that a figure exemplifying the gating strategy is provided in the Supplementary Information.

Magnetic resonance imaging

Experimental design

Design type	*Indicate task or resting state; event-related or block design.*
Design specifications	*Specify the number of blocks, trials or experimental units per session and/or subject, and specify the length of each trial or block (if trials are blocked) and interval between trials.*
Behavioral performance measures	*State number and/or type of variables recorded (e.g. correct button press, response time) and what statistics were used to establish that the subjects were performing the task as expected (e.g. mean, range, and/or standard deviation across subjects).*

Acquisition

Imaging type(s)	*Specify: functional, structural, diffusion, perfusion.*
Field strength	*Specify in Tesla*
Sequence & imaging parameters	*Specify the pulse sequence type (gradient echo, spin echo, etc.), imaging type (EPI, spiral, etc.), field of view, matrix size, slice thickness, orientation and TE/TR/flip angle.*
Area of acquisition	*State whether a whole brain scan was used OR define the area of acquisition, describing how the region was determined.*

Diffusion MRI ☐ Used ☐ Not used

Preprocessing

Preprocessing software	*Provide detail on software version and revision number and on specific parameters (model/functions, brain extraction, segmentation, smoothing kernel size, etc.).*
Normalization	*If data were normalized/standardized, describe the approach(es): specify linear or non-linear and define image types used for transformation OR indicate that data were not normalized and explain rationale for lack of normalization.*
Normalization template	*Describe the template used for normalization/transformation, specifying subject space or group standardized space (e.g. original Talairach, MNI305, ICBM152) OR indicate that the data were not normalized.*
Noise and artifact removal	*Describe your procedure(s) for artifact and structured noise removal, specifying motion parameters, tissue signals and physiological signals (heart rate, respiration).*
Volume censoring	*Define your software and/or method and criteria for volume censoring, and state the extent of such censoring.*

Statistical modeling & inference

Model type and settings	*Specify type (mass univariate, multivariate, RSA, predictive, etc.) and describe essential details of the model at the first and second levels (e.g. fixed, random or mixed effects; drift or auto-correlation).*
Effect(s) tested	*Define precise effect in terms of the task or stimulus conditions instead of psychological concepts and indicate whether ANOVA or factorial designs were used.*

Specify type of analysis: ☐ Whole brain ☐ ROI-based ☐ Both

Statistic type for inference	*Specify voxel-wise or cluster-wise and report all relevant parameters for cluster-wise methods.*

(See Eklund et al. 2016)

Correction

Describe the type of correction and how it is obtained for multiple comparisons (e.g. FWE, FDR, permutation or Monte Carlo).

Models & analysis

n/a	Involved in the study
☐	☐ Functional and/or effective connectivity
☐	☐ Graph analysis
☐	☐ Multivariate modeling or predictive analysis

Functional and/or effective connectivity

Report the measures of dependence used and the model details (e.g. Pearson correlation, partial correlation, mutual information).

Graph analysis

Report the dependent variable and connectivity measure, specifying weighted graph or binarized graph, subject- or group-level, and the global and/or node summaries used (e.g. clustering coefficient, efficiency, etc.).

Multivariate modeling and predictive analysis

Specify independent variables, features extraction and dimension reduction, model, training and evaluation metrics.
